# Treatment algorithm in patients with ovarian cancer

**Published:** 2020-10-08

**Authors:** I Vergote, H Denys, J De Greve, C Gennigens, K Van De Vijver, J Kerger, P Vuylsteke, JF Baurain

**Affiliations:** Department of Oncology, University Hospitals Leuven, Herestraat 49, 3000 Leuven, Belgium; Department of Medical Oncology, Ghent University Hospital, C. Heymanslaan 10, 9000 Ghent, Belgium; Department of Medical Genetics, University Hospitals Brussels, Laarbeeklaan 101, 1090, Brussels, Belgium; Department of Medical Oncology, Liège University Hospital, Avenue de l’Hôpital 1, 4000 Liège, Belgium; Department of Pathology, Ghent University Hospital, C. Heymanslaan 10, 9000 Ghent, Belgium; Department of Pathological Anatomy, Antwerp University Hospital, Universiteitsplein 1, 2610 Wilrijk, Belgium; Jules Bordet Institute, Brussels University Hospital,Boulevard de Waterloo 121, 1000 Brussels, Belgium; Department of Oncology, UCLouvain, CHU UCL Namur Hospital, Site St-Elisabeth, Place Louise Godin 15, 5000 Namur, Belgium; Department of Oncology, St.-Luc University Hospital Brussels, Avenue Hippocrate 10, 1200 Woluwe-Saint-Lambert, Belgium.

**Keywords:** Epithelial ovarian cancer, treatment algorithm, genetic testing, targeted agents

## Abstract

Most ovarian cancer patients are diagnosed only at advanced stages when survival outcomes are worse, andwhen therapeutic decisions might prove challenging. The fundamental treatment for women with ovarian cancerincludes debulking surgery whenever possible and appropriate systemic therapy (chemotherapy, targeted andantiangiogenic agents). In the last few years, knowledge about histological and molecular characteristics of ovariancancer subtypes and stages has increased considerably. This has enabled the development and improvement ofseveral options for the diagnosis and treatment of ovarian cancer in a patient-tailored approach. Accordingly,therapeutic decisions are guided by the characteristics of the patient and the tumour, especially the molecularfeatures of the cancer subtype and disease stage. Particularly relevant are the advances in early genetic testing ofgermline and somatic mutations involved in DNA repair, and the clinical development of targeted agents. In orderto implement the best individual medical strategies, in this article, we present an algorithm of treatment options,including recently developed targeted agents, for primary and recurrent ovarian cancer patients in Belgium.

## Introduction

Ovarian cancer is the fifth leading cause of cancer-related deaths among women in Europe and theUnited States of America, and is the most frequentcause of death among all gynaecological cancersin Europe (Ferlay et al., 2018; American Cancer Society, 2020a). In 2018, ovarian cancer accounted for 3.7% of all new cancer cases in women in Europe (Ferlay et al., 2018). In Belgium, 752 new cases of ovarian cancer were diagnosed in 2016 (Belgian Cancer Registry, 2016).

Ovarian cancer is often asymptomatic, resulting in diagnosis at advanced stages in most patients. This trend is reflected in the Belgian cancer statistics from 2016; 18.9%, 5.6%, 28.7%, and 25.1% of ovarian cancer patients were diagnosed with stage I,II, III, and IV disease, respectively (21.6% of patients had an unreported stage) (Belgian Cancer Registry, 2016). In Belgium, the five-year relative survival rate (2012‒2016) was 78.0% (95% confidence interval [CI]: 72.0–83.0) in 15- to 44-year-old patients, 52.9% (95%CI: 50.0–55.8) in 45- to 69-year-old patients, and 27.9% (95% CI: 24.8–31.1) in ≥70-year-old patients (Belgian Cancer Registry, 2016). Survival outcomes are worse in the elderly and patients with advanced-stage ovarian cancer at the time of diagnosis. Patients diagnosed with stage I disease had a five-year relative survival rate of 91.0% (survival data in Belgium, 2004–2008) (Belgian Cancer Registry, 2012). In comparison, this rate was only 19.1% among women with stage IV disease at diagnosis.

Here, we aim to provide an overview of the current treatment landscape for ovarian cancer in Belgium to help physicians translate evidence and guidelines into individualised medical strategies for their patients.

## Histological and molecular features of ovarian cancer

Ovarian cancer is a heterogeneous disease: it is comprised of tumours that can show very different morphologies, histologies, grades, and molecular characteristics (Lheureux et al., 2019). Epithelial ovarian cancer (EOC) represents approximately 90% of these malignant tumours, whereas sex-cord stromal, germ cell, and mixed-cell ovarian cancers account for the remaining 10% (Hanby et al., 2003; Rojas et al., 2016).

The term “EOC” does not refer to a single disease but encompasses a group of tumours. According to their morphology, several histological subtypes of EOC have been defined, with the most common being the serous (68%–71%) subtype, followed by clear cell (12%–13%), endometrioid (9%–11%), mucinous (3%), malignant Brenner (1%) and mixed histology (6%) subtypes (Rojas et al., 2016). In a more recent classification, EOCs were divided into five types based on histopathology, immunohistochemistry, and molecular genetic analysis: high-grade serous carcinomas (HGSC; approximately 70%), low-grade serous carcinomas (LGSC; 10%), endometrioid carcinomas (approximately 10%), clear cell carcinomas (approximately 5%), and mucinous carcinomas (<3%) ( Lheureux et al., 2019).The five types of EOC have a distinct tumour biology, which directly affects their prognosis and outcome (Lheureux et al., 2019). Predictive biomarkers (histology and molecular genomics) have been recently identified for each type of EOC (Table I) (Lheureux et al., 2019). Molecular criteria may provide insight for therapy selection by stratifying low-grade diseases into separate clusters; however, the high-grade disease is less genetically defined (Hirst et al., 2017).

**Table I:** Subtypes of epithelial ovarian cancer: histological, molecular and gene mutation characteristics (adapted from Lheureux et al., CA Cancer J Clin 2019;69:280-304).

Epithelial ovarian cancer (about 90% of all ovarian cancers)					
	High-grade serous ovarian cancer	Low-grade serous ovarian cancer	Clear cell	Endometrioid	Mucinous
Occurrence	70% of all EOCs	10% of all EOCs	5% of all EOCs	10% of all EOCs	<3% of all EOCs
Histology	Papillary and solid growth; large mononuclear cells; pleomorphic nuclei with prominent nucleoli and mitotic activity.	Small papillae with cells of uniform nuclei and various amounts of hyalinized stroma; psammoma bodies.	Mixture of tubules, solid areas and complex papillae; cells with prominent nucleoli and clear cytoplasm filled with glycogen.	Cystic or predominantly solid.	Heterogeneous; often composed of benign, borderline, non-invasive, and invasive components.
Molecular aberrations	CNA high TP53 BRCA1/2 CDK12 HRD	CNA low MAPK activation KRAS BRAF NRAS HER2	PI3K/AKT activation RTK/Ras activation ARID1A PI3KCA KRAS PTEN TP53	Wnt/ß-catenin activation PTEN CTNNB1 PPP2R1α PI3KCA ARID1A KRAS TP53	HER2 amplification KRAS
Folatereceptor	Yes	-	-	Yes	-
Hormone receptor	30% PR 80% OR	57% PR 87% OR	8% PR 19% OR	67% PR 76% OR	16% PR 20% OR
MMR deficiency	-	-	Yes	Yes	-

AKT: AKT serine/threonine kinase 1; ARIDA: AT-Rich Interaction Domain 1A; BRAF: carcinoma B-Raf proto-oncogene, serine/ threonine kinase; BRCA1/2: breast cancer 1/2 gene; CDK12: cyclin dependent kinase 12; CNA: copy number alteration; CTNNB1: catenin beta 1; EOC: epithelial ovarian cancer; HER2: human epidermal growth factor receptor 2; HRD: homologous recombina- tion deficiency; KRAS: Kirsten rat sarcoma viral oncogene homologue; MAPK: mitogen-activated protein kinase; MMR: mismatch repair; NRAS: neuroblastoma RAS viral (V-ras) oncogene homologue; OR: oestrogen receptor; PI3K(CA): phosphoinositide-3-ki- nase (catalytic, α polypeptide); PPP2R1α: protein phosphatase 2, subunit A, α isoform; PR: progesterone receptor; PTEN: phosphatase and tensin homologue; RTK: receptor tyrosine kinase; TP53: tumour protein p53; Wnt: wingless-related integration site. Adapted from Lheureux S, Braunstein M, Oza AM. Epithelial ovarian cancer: Evolution of management in the era of precision medicine. CA: A Cancer Journal for Clinicians. 2019;69(4):280-304; distributed under a CC BY-NC 4.0 license [https://doi.org/10.3322/caac.21559]).

Ovarian cancers are staged surgically and pathologically according to two systems: the International Union Against Cancer (UICC) TNM (tumor, node, and metastasis) system of classification of malignant tumours, and the International Federation of Gynecology and Obstetrics (FIGO) staging system (Prat and FIGO Committee on Gynecologic Oncology, 2014; Brierley et al., 2017). FIGO stages are defined by the confinement of the tumour to the ovaries or fallopian tubes (stage I), the involvement of ovaries or fallopian tubes with pelvic extension or primary peritoneal cancer (stage II), the additional cytologically or histologically confirmed spread of the tumour to the peritoneum outside of the pelvis and/or metastasis to the retroperitoneal lymph nodes (stage III), or the confirmation of distant metastasis excluding peritoneal metastases (stage IV) (Prat and FIGO Committee on Gynecologic Oncology, 2014).Ovarian cancer cells may spread to the adjacent genital organs, pelvic peritoneum, pelvic and para-aortic lymph nodes, omentum, organ, and peritoneal surfaces in the upper abdomen and thoracic pleura (Vergote et al., 2016a).

## Genetic testing

Germline or somatic mutations in arrays of genes coding for proteins involved in the homologous recombination (HR) repair of double-strand DNA breaks occur in up to 40–50% of patients with HGSC and more rarely in the other epithelial subtypes (Press et al., 2008; Bell et al., 2011). The most prevalent among these are mutations in the breast cancer susceptibility genes (BRCA1/2), which are clinically validated to be of high value for predictive counselling (if germline), in particular for lifesaving preventive strategies in pre-symptomatic mutation carriers in the family. Indeed, while BRCA1/2 somatic mutations are only found in neoplastic tissue and are thus not passed on to the offspring, germline mutations may be transmitted from parents to their children (Griffiths, 2000).

The homologous recombination deficiency (HRD) diagnosis also has prognostic and therapeutic implications. ABRCA-mutated ovarian cancer has a better prognosis and an increased platinum sensitivity compared to a BRCA-wildtype cancer (Yang et al., 2011). Moreover, in BRCA-mutated ovarian cancer, platinum sensitivity can be preserved over many treatment lines in relapsing patients. BRCA1/2 mutations are synthetically lethal with the inhibition of the poly (ADP-ribose) polymerase (PARP) enzyme: cells harbouring a BRCA loss of function undergo cell death upon PARP inhibition due to an excessive accumulation of unrepaired DNA strand breaks. In normal conditions, the HR and non- homologous end joining (NHEJ) mechanisms repair these breaks (Faraoni and Graziani, 2018). In patients with a BRCA1/2 gene mutational loss-of-function, only the error-prone NHEJ can (incompletely) repair these breaks, resulting in the accumulation of DNA breaks and mutations that lead to cell death by synthetic lethality (Faraoni and Graziani, 2018).

Therefore, the identification of somatic and germline mutations in the BRCA1 and BRCA2 genes (and other HR genes conferring sensitivity to PARP inhibitors [PARPis]) has become an essential guide for treatment decisions (Faraoni and Graziani, 2018).

Among other techniques, next-generation sequencing (NGS) of germline DNA can be used to identify mutations in genes associated with hereditary cancer risk, not only HR genes (Price et al., 2018). A typical panel of such genes are BRCA1/2, mutLhomologue 1 (MLH1), mutS homologue 2 (MSH2), mutS homologue 6 (MSH6), PMS1 homologue 2, mismatch repair system component (PMS2), epithelial cell adhesion molecule (EPCAM), tumour protein p53 (TP53), phosphatase and tensin homologue (PTEN) and serine/threonine kinase 11 (STK11) (Weissman et al., 2012; Silva et al., 2014; Minion et al., 2015). Rarely mutated ovarian cancer predisposing HR genes (PALB2, BRIP1, RAD51C, RAD51D, and BARD1) are also often included in germline panel testing (Norquist et al., 2016).

In Belgium, the Personalised Medicine Commission (ComPerMed) recommends genetic testing for all patients with invasive EOC (excluding borderline or mucinous ovarian cancers), with fallopian tube cancer and with peritoneal cancer, ideally at diagnosis (Personalised Medicine Commission, 2019a; Personalised Medicine Commission, 2019b). NGS testing of the mutational BRCA1/2 status is recommended on both tumour DNA and germline DNA (peripheral blood cells), given that most of the BRCA1/2 mutations detected in tumours are germline (Gadducci et al., 2019; Personalised Medicine Commission, 2019b). In patients without germline or somatic BRCA1/2 mutations in recurrence setting, the Personalised Medicine Commission recommends testing the other HRD and other ovarian cancer risk genes to identify patients eligible for clinical trials and cancer genetic counselling in the relatives. An optimal molecular test for comprehensively detecting the HRD status of cancers still needs to be defined (Personalised Medicine Commission, 2019a; Personalised Medicine Commission, 2019b).

The implementation of early systematic germline testing in ovarian cancer requires workflow changes in health care practices. While current standard clinical practice in many countries implies a pre- test consultation with a qualified geneticist and subsequent BRCA1/2 mutation status testing (Capoluongo, 2016), it has been recently proposed that trained specialised staff at oncology units should be enabled to perform the intake and pre-test genetic counselling (Capoluongo, 2016; Capoluongo et al., 2017). One way to guarantee 100% test rates for all patients with ovarian cancer upon diagnosis would be to use “reflex” (i.e., guaranteed) tumour BRCA1/2 mutation testing as part of the pathology department work-up (Hoskins, 2018). This reflex testing requires informed consent that informs the patient on the possibility of finding germline mutations. With this adapted approach, and if turnaround time can be reduced to a maximum of three weeks, delays in proposing personalised treatment schedules could be avoided. If the test detects a potential germline mutation, the patient should be referred to a cancer geneticist for further counselling (Vergote et al., 2016b). As laparoscopic and surgical samples might be rapidly depleted and sufficient tissue is required to ensure good quality of testing, it remains key to maximally use the specimens (Aisner et al., 2016).

## Treatment landscape

Fundamental principles of treatment for women with ovarian cancer include surgery to reduce the tumour bulk to no residual disease whenever possible and appropriate systemic treatment based on the ovarian cancer subtype, disease stage, biology, and patient characteristics (Lheureux et al., 2019).

## Surgery

### First-line

Primary debulking surgery, which aims at complete resection (i.e., no residual disease), is a cornerstone of ovarian cancer therapy (Lheureux et al., 2019). Optimal primary debulking surgery is significantly associated with prolonged survival (Dogan et al., 2013). Primary surgery is also indispensable for accurate staging of ovarian cancer according to the FIGO and TNM systems (Prat and FIGO Committee on Gynecologic Oncology, 2014).The primary surgical procedure comprises an exploration of the abdomen and pelvis, which is usually followed by a total abdominal hysterectomy, a bilateral salpingo- oophorectomy, and an omentectomy in case of total respectability (Lheureux et al., 2019).

In women with poor general health, a high perioperative risk profile, or a low likelihood of achieving effective cytoreduction because of extensive disease (advanced stages), neo-adjuvant chemotherapy to reduce the tumour burden should precede surgery (Lheureux et al., 2019). The neo- adjuvant chemotherapy should be followed by interval debulking surgery when complete resection or effective reduction to < 1cm disease is achievable (Lheureux et al., 2019). A recent pooled analysis of the long-term follow-ups of two randomised trials has validated this strategy in patients with a high tumour burden at presentation or poor performance status (Vergote et al., 2018).

### Recurrence

In women with recurrent ovarian cancer, the benefit of secondary cytoreductive surgery depends on the patient population; its role remains controversial and may depend on the systemic treatment given (Dogan et al., 2013). In the recent Gynaecologic Oncology Group (GOG) 0213 study, secondary surgical cytoreduction followed by platinum- based chemotherapy did not result in longer overall survival (OS) than chemotherapy alone in patients with platinum-sensitive recurrent ovarian cancer (Coleman et al., 2019b). In this study, 84% of patients received platinum-based chemotherapy with bevacizumab followed by bevacizumab maintenance. In contrast, interim findings from the ongoing third Descriptive Evaluation of preoperative Selection KriTeria for OPerability in recurrent ovarian cancer (DESKTOP III) study showed that secondary surgical cytoreduction resulted in clinically meaningful increases of progression-free survival (PFS) and median time to start the first subsequent therapy in patients with a first relapse of ovarian cancer and a positive German Gynaecological Oncology Group (AGO) score (Performance Status Eastern Cooperative Oncology Group [PS ECOG] 0, ascites ≤500 ml, and complete resection at initial surgery) (Du Bois et al., 2017).Findings from these two studies suggest that secondary cytoreductive surgery may only be beneficial for patients with platinum-sensitive recurrence when complete resection is achievable (Du Bois et al., 2017; Bommert et al., 2018).

## Systemic treatments

### First-line

The current standard first-line systemic treatment, administered after primary debulking surgery or as neoadjuvant therapy is a platinum-based doublet intravenous chemotherapy (carboplatin and paclitaxel, every 3 weeks, or carboplatin every 3 weeks and paclitaxel weekly) with bevacizumab (Lheureux et al., 2019).

In case of intolerance to paclitaxel, alternative platinum-based doublet regimens are carboplatin with docetaxel or with pegylated liposomal doxorubicin (PLD),which should be used according to the patient’s/physician’s treatment preferences and the patient-specific drug tolerability since each of the regimens is associated with a specific toxicity profile (Katsumata, 2003; Pignata et al., 2011).The carboplatin with PLD regimen is not reimbursed in Belgium in first-line.

### Recurrence

Although surgical resection followed by first-line platinum‐based chemotherapy leads to complete remission in many patients, recurrences occur in up to 70% of cases (Dogan et al., 2013).Although the recurrent disease is incurable, treatments in this setting have the intention of controlling the symptoms and the disease, to maintain the quality of life, and to prolong the treatment-free periods. Depending on the duration of the treatment-free interval before disease recurrence, patients are classically categorised into “platinum-sensitive”(relapse ≥ six months since last platinum-based treatment) versus “platinum- resistant” (relapse < six months since last platinum- based treatment) (Buechel et al., 2019). Given the advances in ovarian cancer surveillance allowing for faster detection of relapse, the lack of consensus on how progression is defined, and the emergence of other first-line therapies, new guidelines do not adopt such a stringent six-month limit for defining platinum-sensitive patients (Buechel et al., 2019). Also, more patients may be incorrectly excluded from platinum-based regimens in the future as recurrences will be detected earlier (Buechel et al., 2019).

The management of recurrent ovarian cancer is less validated than that of newly diagnosed ovarian cancer (Dogan et al., 2013).After recurrence, platinum-resistant patients should receive non- platinum-based single-agent chemotherapy (PLD, paclitaxel, topotecan or gemcitabine) (Ledermann et al., 2018). In contrast, platinum-sensitive patients may be re-challenged with platinum-based doublet chemotherapy (Ledermann et al., 2018; Lheureux et al., 2019). The intercalation of a non-platinum regimen could confer a clinically meaningful benefit to some patients with platinum-sensitive relapsed ovarian cancer and give them time to recover from platinum-related toxicities (Colombo, 2017; Romero et al., 2019). A randomised phase III study (ET743-OVA-301) demonstrated improved efficacy with an acceptable tolerance of the association of trabectedin (an antineoplastic agent) plus PLD versus PLD alone in patients with platinum- sensitive relapsed ovarian cancer (Monk et al., 2010), leading to the approval of this association by the European Medicines Agency (EMA) for the treatment of platinum-sensitive relapsed ovarian cancer (European Medicines Agency). Another recent study showed a clinically relevant survival benefit in platinum-sensitive patients with germline BRCA1/2 mutations who were treated in third- line with the association of trabectedin plus PLD compared to PLD alone (Monk et al., 2020).

### Targeted and antiangiogenic agents

Treatment of first-line and recurrent ovarian cancer has now expanded to include targeted and antiangiogenic agents (Lheureux et al., 2019).The targeted agents approved by the EMA include three PARPis (olaparib, niraparib and conditional approval for rucaparib) (European Medicines Agency, 2019a; European Medicines Agency, 2019b; European Medicines Agency, 2019c). In addition, one antiangiogenic agent (bevacizumab) is also approved by the EMA (European Medicines Agency, 2019d). Pivotal trials evaluating their use in first- and further-line treatment are detailed in Tables II and III.

**Table II:** Pivotal clinical trials of first-line treatment with targeted agents in ovarian cancer patients.

Trial	Number of patients	Treatment, first line	Patient population	Homologous recombination repair gene mutation status	PFS	HR	OS	HR	Adverse events
SOLO1 (Moore et al. 2018) (NCT01844986)	391	Maintenance monotherapy with olaparib or placebo	Newly diagnosed advanced (FIGO stage III or IV) high-grade serous or endometrioid ovarian cancer, primary peritoneal cancer, or fallopian-tube cancer (or a combination thereof)	*BRCA* mutation	At 41 months of follow-up, the median PFS for patients treated with olaparib was not reached, compared to 13.8 months for patients treated with placebo	HR for disease progression or death: 0.30 (95% CI: 0.23–0.41; p<0.001)	-	-	Adverse events were consistent with the known toxic effects of olaparib; the most frequent were nausea, fatigue and vomiting. Higher frequency of grade ≥3 anaemia and neutropenia in the olaparib versus placebo group
ICON7 (Perren et al. 2011) (NCT00483782)	1528	Carboplatin and paclitaxel given every 3 weeks for 6 cycles with or without bevacizumab given concurrently every 3 weeks for 5 to 6 cycles and continued for 12 additional cycles or until disease progression	Histologically confirmed, high-risk, early-stage disease (FIGO stage I or IIA) or advanced (FIGO stage IIB to IV) epithelial ovarian cancer, primary peritoneal cancer, or fallopian-tube cancer	-	At 36 months, PFS was 20.3 months with standard therapy, as compared to 21.8 months with standard therapy plus bevacizumab	HR for disease progression or death with bevacizumab added: 0.81 (95% CI: 0.70–0.94; p=0.004)	-	-	Bevacizumab was associated with more toxic effects (most often hypertension of grade ≥2: 18% versus 2% with chemotherapy alone)
PAOLA-1 (Ray-Coquard et al. 2019) (NCT02477644)	806	Maintenance monotherapy with olaparib plus bevacizumab or bevacizumab alone	Newly diagnosed FIGO stage III–IV high-grade serous or endometrioid ovarian cancer, fallopian tube or primary peritoneal cancer. Patients had received first-line standard platinum-based chemotherapy plus bevacizumab and were in clinical complete or partial response	Overall population	PFS was 22.1 months with olaparib plus bevacizumab and 16.6 months in bevacizumab alone	HR for disease progression or death with olaparib added: 0.59 (95% CI: 0.49–0.72; p<0.001)	-	-	-
				*BRCA* mutation	PFS was 37.2 months with olaparib plus bevacizumab and 21.7 months in bevacizumab alone	HR for disease progression or death with olaparib added: 0.31 (95% CI: 0.20–0.47)			
PRIMA (Gonzalez-Martin et al. 2019) (NCT02655016)	733	Maintenance monotherapy with niraparib or placebo once daily in 28-day cycles for 36 months or until disease progression	Newly diagnosed FIGO stage III–IV, advanced cancer of the ovary, peritoneum, or fallopian tube who had received six to nine cycles of first-line platinum-based chemotherapy that resulted in a complete or partial response	Overall population	PFS was 13.8 months in the niraparib group versus 8.2 months in the placebo group	HR for disease progression or death: 0.62 (95% CI: 0.50–0.76; p<0.001)	At 24 months: 84% in the niraparib group versus 77% in the placebo group	HR: 0.70 (95% CI: 0.44–1.11)	Most common adverse events of grade 3 or higher were anaemia (in 31.0% of the patients), thrombocytopenia (in 28.7%), and neutropenia (in 12.8%) Higher frequency of myelosuppression and low-grade nausea in the niraparib group than in the placebo group
				Homologous-recombination deficiency*	PFS was 21.9 months in the niraparib group versus 10.4 months in the placebo group	HR for disease progression or death: 0.43 (95% CI: 0.31–0.59; p<0.001)	At 24 months: 91% in the niraparib group versus 85% in the placebo group	HR: 0.61 (95% CI: 0.27–1.39)	
VELIA (Coleman et al. 2019a) (NCT02470585)	1140	Chemotherapy plus veliparib followed by veliparib maintenance (veliparib-throughout group); chemotherapy plus veliparib followed by placebo maintenance (veliparib-combination-only group); chemotherapy plus placebo followed by placebo maintenance (control group)	Previously untreated FIGO stage III or IV high-grade serous epithelial ovarian, fallopian tube, or primary peritoneal carcinoma	Overall population	PFS was 23.5, 15.2 and 17.3 in the veliparib-throughout, the veliparib-combination-only, and the control groups, respectively	HR for disease progression or death: in veliparib-throughout versus control: 0.68 (95% CI: 0.56–0.83; P<0.001); in veliparib-combination-only versus control: 1.07 (95% CI: 0.90–1.29) in the intention-to-treat cohort	-	-	Veliparib was associated to a higher incidence of anaemia and thrombocytopenia when combined with chemotherapy, and to nausea and fatigue overall
				Homologous-recombination deficiency*	PFS was 31.9, 18.1 and 20.5 months in the veliparib-throughout, the veliparib-combination-only, and the control groups, respectively	HR for disease progression or death: in veliparib-throughout versus control: 0.57 (95% CI: 0.43–0.76; p<0.001); in veliparib-combination-only versus control: 1.10 (95% CI: 0.86–1.41)	-	-	
				*BRCA* mutation	PFS was 34.7, 21.1 and 22.0 months in the veliparib-throughout, the veliparib-combination-only, and the control groups, respectively	HR for disease progression or death: in veliparib-throughout versus control: 0.44 (95% CI: 0.28–0.68; p<0.001); in veliparib-combination-only versus control: 1.22 (95% CI: 0.82–1.80)	-	-	

BRCA: breast cancer gene; CI: confidence interval; FIGO: International Federation of Gynecology and Obstetrics; HR: hazard ratio; OS: overall survival; PFS: progression-free survival. *Defined as the presence of a BRCA deleterious mutation, a score of at least 42 on the my-Choice test, or both.

**Table III:** Pivotal clinical trials of second-line treatment with targeted agents in ovarian cancer patients.

Trial	Number of patients	Treatment, second or further line	Patient population	Homologous recombination repair gene mutation status	PFS	HR	OS	HR	Adverse events
Study 19 (Ledermann et al. 2014) (NCT00753545)	265	Maintenance monotherapy with olaparib versus placebo	Platinum-sensitive recurrent serous ovarian cancer	*BRCA* mutation	11.2 months (95% CI, 8.3–not calculable) versus placebo 4.3 months (3.0–5.4)	0.18 (0.10–0.31); p<0.0001	34.9 months (95% CI, 29.2–not calculable versus placebo 31.9 months (23.1–40.7)	0.73 (95% CI 0.45–1.17); p=0.19	Grade 3 or worse: fatigue (in ten [7%] patients in the olaparib group versus four [3%] in the placebo group) and anaemia (seven [5%] versus one [<1%])
				No *BRCA* mutation	7.4 months (5.5–10.3) versus placebo 5.5 months (3.7–5.6)	0.54 (0.34–0.85); p=0.0075	24.5 months (19.8–35.0) versus placebo 26.2 months (22.6–33.7)	0.99 (95% CI 0.63–1.55); p=0.96	
SOLO2/ENGOT-Ov21 (Pujade-Lauraine et al. 2017) (NCT01874353)	295	Maintenance monotherapy with olaparib versus placebo	Platinum-sensitive, recurrent high-grade serous ovarian cancer or endometrioid cancer	*BRCA* mutation	19.1 months (95% CI, 16.3–25.7) versus placebo 5.5 months (5.2–5.8)	0.30 (95% CI 0.22–0.41)			Grade 3 or worse: (38 [19%] of 195 patients in the olaparib group versus two [2%] of 99 patients in the placebo group), fatigue or asthenia (eight [4%] versus two [2%]), and neutropenia (ten [5%] versus four [4%])
NOVA (Mirza et al. 2016) (NCT01847274)	553	niraparib (300 mg) or placebo once daily until disease progression	Platinum-sensitive recurrent, ovarian, fallopian tube, or peritoneal cancer	Germline *BRCA* mutation	21.0 months compared with 5.5 months in the placebo group	0.27 (95% CI, 0.17 to 0.41); P<0.001			Most common grade 3 or 4 adverse events that were reported in the niraparib group were thrombocytopenia (in 33.8%), anaemia (in 25.3%), and neutropenia (in 19.6%)
				No germline *BRCA* mutation but with homologous recombination deficiency	12.9 months versus 3.8 months in the placebo group	0.38 (95% CI, 0.24 to 0.59); P<0.001			
				No germline *BRCA* mutation	9.3 months versus 3.9 months in the placebo group	0.45 (95% CI, 0.34 to 0.61); P<0.001			
NOVA subgroup analysis in patients aged ≥70 years (Fabbro et al. 2019) (NCT01847274)	95 (≥70 years)	Niraparib (300 mg) or placebo once daily until disease progression	Platinum-sensitive recurrent, ovarian, fallopian tube, or peritoneal cancer	Germline *BRCA* mutation	Not reached versus placebo 3.7 months	0.09 (95% CI, 0.01 to 0.73)			Grade 3 or worse: thrombocytopenia event (34.4%), anaemia event (13.1%), and neutropenia event (16.4%)
				No germline *BRCA* mutation	11.3 months compared with placebo 3.8 months	0.35 (95% CI, 0.18 to 0.71)			
ARIEL2 (Swisher et al. 2017) (NCT01891344)	206	Oral rucaparib 600 mg twice daily or placebo	Platinum-sensitive, recurrent, high-grade epithelial ovarian, fallopian tube, or primary peritoneal cancer	*BRCA* mutation	12.8 months (95% CI, 9.0–14.7)	0.27 (95% CI 0.16–0.44, p<0.0001)			Most common grade 3 or worse treatment-emergent adverse events were anaemia or decreased haemoglobin (45 [22%] patients), and elevations in alanine aminotransferase or aspartate aminotransferase (25 [12%]).
				*BRCA* mutant or *BRCA* wild-type and high LOH	5.7 months (95% CI, 5.3–7.6)	0.62 (0.42–0.90, p=0.011) compared to low LOH group			
				*BRCA* wildtype and low/intermediated LOH	5.2 months (95% CI, 3.6–5.5)				
ARIEL3(Coleman et al. 2017) (NCT01968213)	564	Oral rucaparib 600 mg twice daily or placebo in 28-day-cycles	Platinum-sensitive, high-grade serous or endometrioid ovarian, primary peritoneal, or fallopian tube carcinoma	*BRCA* mutation	16.6 months (95% CI, 13.4–22.9) versus 5.4 months (3.4–6.7)	0.23 (95% CI, 0.16–0.34); p<0.0001			Grade 3 or worse: anaemia or decreased haemoglobin concentration (70 [19%] patients versus one [1%]) and increased alanine or aspartate aminotransferase concentration (39 [10%] versus none)
				*BRCA* mutant or *BRCA* wild-type and high LOH	13.6 months (10.9–16.2) versus 5.4 months (5.1–5.6)	0.32 (0.24–0.42); p<0.0001			
				*BRCA* wildtype and low/intermediated LOH	0.8 months (8.3–11.4) versus 5.4 months (5.3–5.5)	0.36 (0.30–0.45); p<0.0001			

BRCA: breast cancer gene; CI: confidence interval; HR: hazard ratio; LOH: loss of heterozygosity; OS: overall survival; PFS: progression-free survival.

**Figure 1:**
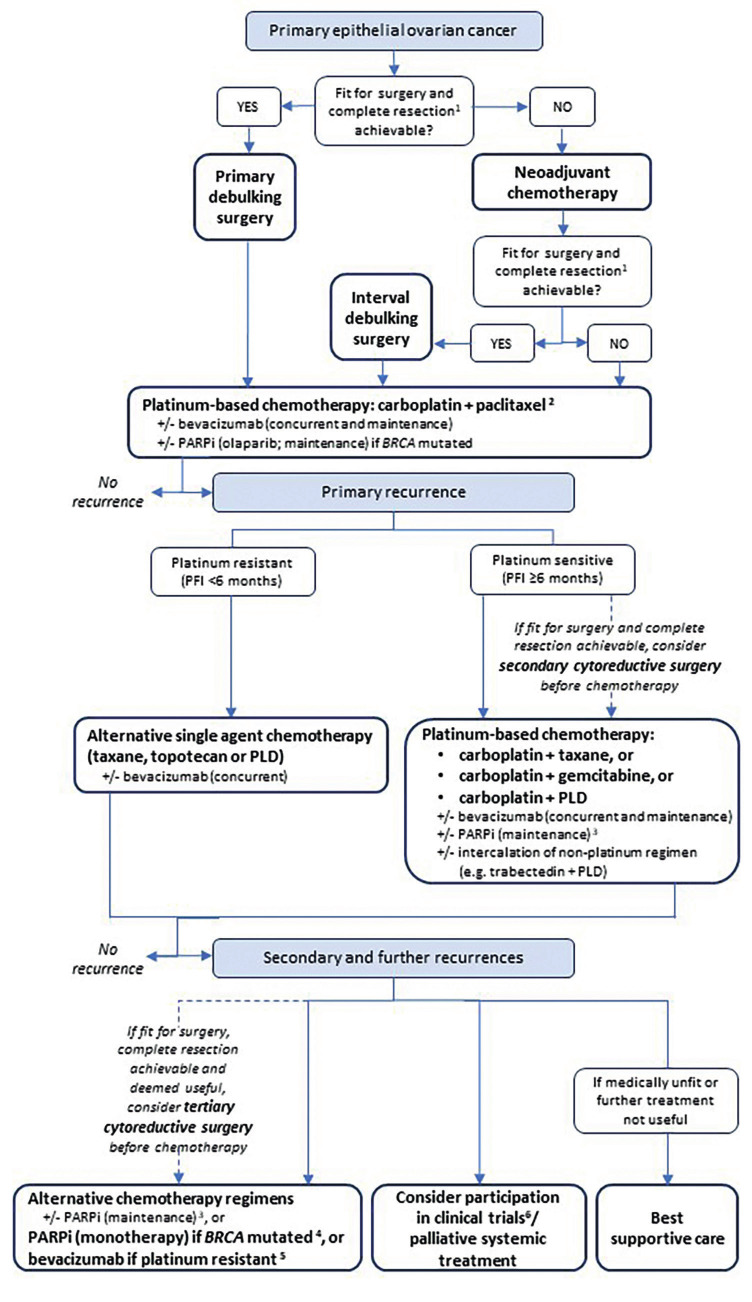
Treatment algorithm for epithelial ovarian cancer. +/-: with/without; debulking: complete resection (i.e., residual disease=0 cm); BRCA: breast cancer gene; PARPi: poly (ADP-ribose) polymerase inhibitor; PFI: platinum-free interval until relapse; PLD: pegylated liposomal doxorubicin. 1 The goal is complete resection, but surgery is sometimes indicated if only effective cytoreduction (<1 cm of residual disease) is achievable. 2 If intolerance to paclitaxel, alternative regimens should be used ac- cording to preference and tolerability (carboplatin + docetaxel or carboplatin + PLD). 3 Olaparib, niraparib and rucaparib (niraparib and rucaparib are not reimbursed in Belgium yet). 4 In Europe, only rucaparib is licensed by the European Medicines Agencyas a monotherapy for patients with platinum-sensitive disease. 5 For patients who have not received prior therapy with a vascular endothelial growth factor receptor-targeted agent. 6 Participation in clinical trials is in all steps of the flowchart an option.

In Europe, olaparib, niraparib and rucaparib are indicated as monotherapy for the maintenance treatment of adult patients with platinum-sensitive, relapsed, high-grade, serous epithelial ovarian, fallopian tube, or primary peritoneal cancer (BRCA- mutated for olaparib) who are in response to platinum-based chemotherapy (European Medicines Agency, 2019a; European Medicines Agency, 2019b; European Medicines Agency, 2019c). Rucaparib is also indicated as a monotherapy treatment of adult patients with platinum-sensitive, relapsed or progressive, BRCA-mutated, high- grade, epithelial ovarian, fallopian tube, or primary peritoneal cancer who have been treated with at least two prior lines of platinum-based chemotherapy and are unable to tolerate further platinum-based chemotherapy (European Medicines Agency, 2019c). In Belgium, olaparib is reimbursed as a single agent for maintenance treatment in patients with newly diagnosed, advanced ovarian cancer and a BRCA1/2 mutation, who have a complete or partial clinical response after first-line platinum- based chemotherapy. The randomised, double-blind, phase III trial (SOLO1 study) has shown that the use of maintenance therapy with olaparib compared with placebo provides a substantial benefit with regard to PFS in this population (Table II) (Moore et al., 2018).

Moreover, olaparib is reimbursed in Belgium as a single agent for maintenance treatment in patients with recurrent platinum-sensitive, PARP-naïve, BRCA-mutated, high-grade EOC, fallopian tube cancer or primary peritoneal cancer after at least two prior lines of platinum-based chemotherapy (Specenier, 2016). Niraparib and rucaparib are not yet reimbursed for that indication.

Bevacizumab is indicated and reimbursed in Belgium in combination with carboplatin and paclitaxel for the first-line treatment of adult patients with advanced epithelial ovarian, fallopian tube, or primary peritoneal cancer (Neyt et al., 2017; European Medicines Agency, 2019d). Bevacizumabin combined with carboplatin and gemcitabine, or carboplatin and paclitaxel is also indicated and reimbursed in Belgium for the treatment of adult patients with a first recurrence of platinum-sensitive epithelial ovarian, fallopian tube or primary peritoneal cancer, who have not received prior therapy with bevacizumab or other vascular endothelial growth factor (VEGF) inhibitors or VEGF receptor-targeted agents. A third approved indication, which is also reimbursed in Belgium, is bevacizumab in combination with paclitaxel, topotecan or PLD for the treatment of adult patients with platinum-resistant recurrent disease and who had received no more than two prior chemotherapy regimens and who have not received prior therapy with bevacizumab or other VEGF inhibitors or VEGF receptor-targeted agents.

Clinical trials are evaluating other indications for the above-mentioned targeted therapies and antiangiogenic agents as well as new agents. A phase III, randomised study (PAOLA-1 trial) has shown that the addition of olaparib to bevacizumab maintenance therapy following first-line platinum- based chemotherapy plus bevacizumab led to a statistically significant and clinically meaningful PFS benefit in patients with advanced ovarian cancer; especially in those with a BRCA mutation and in HRD-positive patients (Ray-Coquard et al., 2019). A second phase III, randomised study (PRIMA trial) has shown that patients with newly-diagnosed advanced ovarian cancer who received niraparib after a response to platinum- based chemotherapy had significantly longer PFS than those who received placebo, regardless of the presence or absence of HRD as determined by the my-Choice test (Myriad Genetics) (Gonzalez- Martin et al., 2019). A third phase III, randomised study (VELIA trial) has shown that another PARPi (veliparib) in combination with chemotherapy as initial treatment followed by veliparib maintenance therapy in patients with high-grade serous ovarian carcinoma led to significantly longer PFS than chemotherapy alone (Coleman et al., 2019a).

## Conclusions

Accurate pathological and molecular testing is essential to construct multifactorial, patient-tailored treatment approaches in ovarian cancer. Based on recent results, infrastructural and pragmatic workflow changes are necessary to implement early systematic genetic testing and counselling, and to timely plan the integration of targeted agents and antiangiogenic agents, which have now proven their efficacy in clinical practice, into treatment strategies. Since new treatments for ovarian cancer are currently under development and should be implemented in the coming years, ongoing research should further help refine the precise treatment algorithm and answer open questions in this rapidly evolving field.

## References

[1] Aisner DL, Rumery MD, Merrick DT (2016). Do more with less: tips and techniques for maximizing small biopsy and cytology specimens for molecular and ancillary testing. The University of Colorado Experience. Arch Pathol Lab Med.

[2] Key statistics for ovarian cancer.

[3] Treatment of invasive epithelial ovarian cancers, by stage.

[4] Cancer survival in Belgium, 2004-2008.

[5] Cancer Fact Sheet Ovarian Cancer, 2016.

[6] Bell D, Berchuck A, Birrer M (2011). Integrated genomic analyses of ovarian carcinoma.. Nature.

[7] Bommert M, Harter P, Heitz F (2018). Clin Oncol (R Coll Radiol). When should surgery be used for recurrent ovarian carcinoma.

[8] Brierley JD, Gospodarowicz MK, Wittekind C (2017). TNM Classification of Malignant Tumours.

[9] Buechel M, Herzog TJ, Westin SN (2019). Treatment of patients with recurrent epithelial ovarian cancer for whom platinum is still an option.. Ann Oncol.

[10] Capoluongo E (2016). BRCA to the future: towards best testing practice in the era of personalised healthcare.. Eur J Hum Genet.

[11] Capoluongo E, Ellison G, Lopez-Guerrero JA (2017). Guidance statement on BRCA1/2 tumor testing in ovarian cancer patients.. Semin Oncol.

[12] Coleman RL, Fleming GF, Brady MF (2019). Veliparib with first-line chemotherapy and as maintenance therapy in ovarian cancer.. N Engl J Med.

[13] Coleman RL, Oza AM, Lorusso D (2017). Rucaparib maintenance treatment for recurrent ovarian carcinoma after response to platinum therapy (ARIEL3): a randomised, double-blind, placebo-controlled, phase 3 trial.. Lancet.

[14] Coleman RL, Spirtos NM, Enserro D (2019). Secondary surgical cytoreduction for recurrent ovarian cancer.. N Engl J Med.

[15] Colombo N (2017). When nonplatinum is the answer: the role of trabectedin plus pegylated liposomal doxorubicin in recurrent ovarian cancer.. Future Oncol.

[16] Colombo N, Sessa C, Bois AD (2019). ESMO-ESGO consensus conference recommendations on ovarian cancer: pathology and molecular biology, early and advanced stages, borderline tumours and recurrent disease. Int J Gynecol Cancer.

[17] Dogan NU, Schneider A, Chiantera V (2013). Tertiary cytoreduction in the setting of recurrent ovarian cancer.. Oncol Lett.

[18] Du Bois A, Vergote I, Ferron G (2017). Randomized controlled phase III study evaluating the impact of secondary cytoreductive surgery in recurrent ovarian cancer: AGO DESKTOP III/ENGOT ov20.. J Clin Oncol.

[19] Summary of product characteristics. Yondelis..

[20] Summary of product characteristics. Lynparza..

[21] Summary of product characteristics. Zejula..

[22] Summary of product characteristics. Rubraca..

[23] Summary of product characterisitcs. Avastin..

[24] Fabbro M, Moore KN, Dorum A (2019). Efficacy and safety of niraparib as maintenance treatment in older patients (>/=70years) with recurrent ovarian cancer: Results from the ENGOT-OV16/NOVA trial.. Gynecol Oncol.

[25] Faraoni I, Graziani G (2018). Role of BRCA mutations in cancer treatment with poly(ADP-ribose) polymerase (PARP) inhibitors.. Cancers (Basel).

[26] Ferlay J, Colombet M, Soerjomataram I (2018). Cancer incidence and mortality patterns in Europe: Estimates for 40 countries and 25 major cancers in 2018.. Eur J Cancer.

[27] Gadducci A, Guarneri V, Peccatori FA (2019). Current strategies for the targeted treatment of high-grade serous epithelial ovarian cancer and relevance of BRCA mutational status.. J Ovarian Res.

[28] Gonzalez-Martin A, Pothuri B, Vergote I (2019). Niraparib in patients with newly diagnosed advanced ovarian cancer.. N Engl J Med.

[29] Griffiths AJF, Miller JH, Suzuki DT (2000). An introduction to genetic analysis.

[30] Hanby AM, Walker C, Tavassoli FAE (2003). World Health Organization classification of tumours. Pathology and genetics of tumours of the breast and female genital organs.

[31] Hirst J, Crow J, Godwin A (2017). Ovarian cancer genetics: subtypes and risk factor.

[32] Hoskins PJ (2018). Inadequate Rates of BRCA Testing with its negative consequences for women with epithelial ovarian cancer and their families: an overview of the literature.. Clin Oncol (R Coll Radiol).

[33] Katsumata N (2003). Docetaxel: an alternative taxane in ovarian cancer.. Br J Cancer.

[34] Ledermann J, Harter P, Gourley C (2014). Olaparib maintenance therapy in patients with platinum-sensitive relapsed serous ovarian cancer: a preplanned retrospective analysis of outcomes by BRCA status in a randomised phase 2 trial.. Lancet Oncol.

[35] Ledermann JA, Raja FA, Fotopoulou C (2018). Corrections to “Newly diagnosed and relapsed epithelial ovarian carcinoma: ESMO clinical practice guidelines for diagnosis, treatment and follow-up.. Annals of Oncology.

[36] Lheureux S, Braunstein M, Oza AM (2019). Epithelial ovarian cancer: evolution of management in the era of precision medicine.. CA Cancer J Clin.

[37] Minion LE, Dolinsky JS, Chase DM (2015). Hereditary predisposition to ovarian cancer, looking beyond BRCA1/BRCA2.. Gynecol Oncol.

[38] Mirza MR, Monk BJ, Herrstedt J (2016). Niraparib maintenance therapy in platinum-sensitive, recurrent ovarian cancer.. N Engl J Med.

[39] Monk BJ, Herzog TJ, Kaye SB (2010). Trabectedin plus pegylated liposomal doxorubicin in recurrent ovarian cancer.. J Clin Oncol.

[40] Monk BJ, Herzog TJ, Wang G (2020). A phase 3 randomized, open-label, multicenter trial for safety and efficacy of combined trabectedin and pegylated liposomal doxorubicin therapy for recurrent ovarian cancer.. Gynecol Oncol.

[41] Moore KN, Colombo N, Scambia G (2018). Maintenance olaparib in patients with newly diagnosed advanced ovarian cancer.. N Engl J Med.

[42] Neyt M, Devriese S, Camberlin C (2017). Bevacizumab in the treatment of ovarian cancer. Health Technolog Assessment (HTA) Brussels: Belgian Health Care Knowledge Centre (KCE).

[43] Norquist BM, Harrell MI, Brady MF (2016). Inherited mutations in women with ovarian carcinoma.. JAMA Oncol.

[44] Perren TJ, Swart AM, Pfisterer J (2011). A Phase 3 trial of bevacizumab in ovarian cancer.. N Engl J Med.

[45] Ovarian cancer. Test description.

[46] (Retrieved 20th March, 2020, from https://www). Ovarian cancer. Algorithm.

[47] Pignata S, Scambia G, Ferrandina G (2011). Carboplatin plus paclitaxel versus carboplatin plus pegylated liposomal doxorubicin as first-line treatment for patients with ovarian cancer: the MITO-2 randomized phase III trial.. J Clin Oncol.

[48] Prat J (2014). Staging classification for cancer of the ovary, fallopian tube, and peritoneum.. Int J Gynaecol Obstet.

[49] Press JZ, De Luca A, Boyd N (2008). Ovarian carcinomas with genetic and epigenetic BRCA1 loss have distinct molecular abnormalities.. BMC Cancer.

[50] Price KS, Svenson A, King E (2018). Inherited Cancer in the age of next-generation sequencing.. Biol Res Nurs.

[51] Pujade-Lauraine E, Ledermann JA, Selle F (2017). Olaparib tablets as maintenance therapy in patients with platinum-sensitive, relapsed ovarian cancer and a BRCA1/2 mutation (SOLO2/ENGOT-Ov21): a double-blind, randomised, placebo-controlled, phase 3 trial.. Lancet.

[52] Ray-Coquard IL, Pautier P, Pignata S (2019). Phase III PAOLA-1/ENGOT-ov25 trial: Olaparib plus bevacizumab (bev) as maintenance therapy in patients (pts) with newly diagnosed, advanced ovarian cancer (OC) treated with platinum-based chemotherapy (PCh) plus bev.. Ann Oncol.

[53] Rojas V, Hirshfield KM, Ganesan S (2016). Molecular characterization of epithelial ovarian cancer: implications for diagnosis and treatment.. Int J Mol Sci.

[54] Romero I, Mallol P, Santaballa A (2019). Multicenter retrospective study to evaluate the impact of trabectedin plus pegylated liposomal doxorubicin on the subsequent treatment in women with recurrent, platinum-sensitive ovarian cancer.. Anticancer Drugs.

[55] Silva FC, Lisboa BC, Figueiredo MC (2014). Hereditary breast and ovarian cancer: assessment of point mutations and copy number variations in Brazilian patients.. BMC Med Genet.

[56] Specenier P (2016). New oncology reimbursements in Belgium.. Belg J Med Oncol.

[57] Swisher EM, Lin KK, Oza AM (2017). Rucaparib in relapsed, platinum-sensitive high-grade ovarian carcinoma (ARIEL2 Part 1): an international, multicentre, open-label, phase 2 trial.. Lancet Oncol.

[58] Vergote I, Banerjee S, Gerdes AM (2016). Current perspectives on recommendations for BRCA genetic testing in ovarian cancer patients.. Eur J Cancer.

[59] Vergote I, Coens C, Nankivell M (2018). Neoadjuvant chemotherapy versus debulking surgery in advanced tubo-ovarian cancers: pooled analysis of individual patient data from the EORTC 55971 and CHORUS trials.. Lancet Oncol.

[60] Vergote I, Vlayen J, Heus P (2016). Ovarian cancer: diagnosis, treatment and follow-up. Good Clinical Practice (GCP) Brussels: Belgian Health Care Knowledge Centre (KCE).

[61] Weissman SM, Weiss SM, Newlin AC (2016). Genetic testing by cancer site: ovary.. Cancer J.

[62] Wright AA, Bohlke K, Armstrong DK (2016). Neoadjuvant chemotherapy for newly diagnosed, advanced ovarian cancer: society of gynecologic oncology and American Society of Clinical Oncology clinical practice guideline.. J Clin Oncol.

[63] Yang D, Khan S, Sun Y (2011). Association of BRCA1 and BRCA2 mutations with survival, chemotherapy sensitivity, and gene mutator phenotype in patients with ovarian cancer.. Jama.

